# Supplementary material for: α-Synuclein triggers intercellular nanotubes formation to prevent apoptosis in astroglia by promoting stemness
Source: Cell Biosci. 2026 Jul 5;16:109. doi: 10.1186/s13578-026-01617-9 (PMC13599109; doi:10.1186/s13578-026-01617-9)
Supplement: Supplementary file 2 [file 13578_2026_1617_MOESM2_ESM.docx]

**Supplementary Materials for Full WBs**

**α-Synuclein Triggers Intercellular Nanotubes Formation to Prevent Apoptosis in Astroglia by Promoting Stemness**

Rachana Kashyap^1^, Anirudh Sreenivas BK^1^, Varshith MR^2^, Rajashri Rameshwar Mundada^1^, Sreedevi P^3^, Shreshta Jain^1^, Archanalakshmi Kambaru^4^, Somasish Ghosh Dastidar^2^, Sivaraman Padavattan^4^, Vinay Kumar Rao^1^, Ravi Manjithaya^3^, Jiri Neuzil^5,6^, Sangeeta Nath^1*^.

^1^Manipal Institute of Regenerative Medicine, Bangalore, Manipal Academy of Higher Education, Manipal, India

^2^ Centre for Molecular Neurosciences, Kasturba Medical College, Manipal Academy of Higher Education, Manipal, India

^3^ Autophagy Laboratory, Molecular Biology and Genetics Unit, Jawaharlal Nehru Centre for Advanced Scientific Research, Bengaluru, India.

^4^ Department of Biophysics, National Institute of Mental Health and Neurosciences, Bangalore, India.

^5^Institute of Biotechnology, Czech Academy of Sciences, Prague-West, Czech Republic.

^6^School of Pharmacy and Medical Science, Griffith University, Southport, QLD, Australia.

*Correspondence to be addressed [sangeeta.nath@manipal.edu](mailto:sangeeta.nath@manipal.edu)

**Key words:** α- synuclein, tunneling nanotubes, mitochondria transfer, reactive oxygen species (ROS), astroglia, cancer stem-like cells, apoptosis, p53-degradation

***Figure S5:*** *Full WBs of A) Figure 3H (Histone H3 and Nanog). Nanog blot was developed after stripping Histone H3 bands. In the stripped blot, Mw markers are not visible. B) Full blot of Figure 3H (Phospho-Nanog and Histone H3). Blot was cut at below 25 kDa,, then, Phospho-Nanog and Histone H3 bands were developed separately. C) Nuclear fraction samples were developed with GAPDH and Histone H3, and results validate the absence of cytosolic protein GAPDH.*

***Figure S6:*** *Full WBs of A) Figure 5I (p53, p21 and actin). B) Full blots of Figure 5H (Nanog and actin). C) Full blots of Figure 5KM (LC3B and Actin). D) Full blots of Figure 5L, Ub (ubiquitinated p53, p53 input blot and loading control Actin.*

A

B

***Figure S7:*** *Full WBs of A) Figure 6O, Caspase-3 in shNanog knockdown cells and pLKO.1 control cells after 24 hours, with and without 1μM α-SYN protofibril treatment. B) Full blots of Figure 7J, Caspase-3 in U87 MG cells after 24 hours of α-SYN protofibril (1μM) and Y-27632* (5μM),  *with and without actin-cytoskeleton inhibitor Cyto-D inhibitor* (0.5μM)*. Blots highlight pro-Caspase and cleaved-Caspase-3 bands, and GAPDH as a loading control.*
